# Seasonal variation in mineralization rates (C-N-P-Si) of mussel *Mytilus edulis* biodeposits

**DOI:** 10.1007/s00227-012-1944-3

**Published:** 2012-05-13

**Authors:** H. M. Jansen, M. C. J. Verdegem, Ø. Strand, A. C. Smaal

**Affiliations:** 1 Institute of Marine Research (IMR), Nordnesgaten 50, 5817 Bergen, Norway; 2 Wageningen Institute for Marine Resources and Ecosystem Studies (IMARES), Korringaweg 5, 4401 NT Yerseke, The Netherlands; 3 Department of Aquaculture and Fisheries, Wageningen University, Marijkeweg 40, 6709 PG Wageningen, The Netherlands

## Abstract

To determine seasonal variability in mineralization dynamics of mussel biodeposits, we applied a multiple-element approach measuring mineralization rates of carbon (C), nitrogen (N), phosphorus (P) and silicate (Si) during three periods (March, August and November). The results of this study showed that mineralization rates vary between seasons and between elements and that mineralization dynamics were influenced by both temperature and biodeposit nutrient composition. Mineralization rates were 3.2 ± 0.4 mmol C, 0.17 ± 0.04 mmol N, 0.06 ± 0.02 mmol P and 3.91 ± 3.75 mmol Si per gram biodeposit (DW) per day, which represented 24 % of the particulate organic C and 17 % of the particulate organic N in mussel biodeposits. Seasonal variability was largest for Si mineralization with 60–80-fold higher rates measured in March compared to August and November. This difference is most likely related to the difference in biodeposit nutrient composition. It was furthermore shown that the labile fraction of biodeposits became mineralized after, respectively, 18, 9 and 13 days during the experimental periods in March, August and November. This indicates that temperature enhances biodeposit decomposition with approximately 2–3 times faster turnover at a 10 °C temperature interval (*Q*_*10*_).

## Introduction

Suspension feeding mussels have the potential to filter considerable quantities of particulate matter from the water column (Cranford et al. 2011). Ingested food particles are digested, and the remnants, together with some metabolic waste products, are expelled as feces (Hawkins and Bayne 1985). During periods of excess food availability, mussels reject part of the filtered material before ingestion and expel it from the inhalant siphon as pseudofeces (Ward and Shumway 2004). Feces and pseudofeces are collectively called biodeposits. Mussel biodeposits are rich in organic nutrients (Kaspar et al. 1985; Grenz et al. 1990) and show relatively high decay rates compared to decomposing phytoplankton or macroalgae (Giles and Pilditch 2006). Although measurements on biodeposit degradation are essential to understand and quantify bivalve-ecosystem interactions, relatively little has been published on bivalve biodeposit quality and specific mineralization and decay rates (see review by McKindsey et al. 2011). The ecological importance of biodeposit mineralization is the availability of regenerated nutrients for primary producers (Prins et al. 1998).

Decomposition of organic material (including mussel biodeposits) is often described by first-order (G) or multi-G exponential models (Canfield et al. 2005), which roughly can be divided into two phases: (1) a steep decrease in decomposition rates during the first 4–8 days after deposition, indicating that the labile nutrient pool in biodeposits becomes depleted (Giles and Pilditch 2006; Carlsson et al. 2010) and (2) a period with very low decomposition rates representing the refractory material that decomposes on much longer time scales. Yet, all organic material (OM) will eventually be mineralized (Meyerreil 1994). It is suggested that high mineralization rates of biodeposits are related to the presence of resident gut bacteria that are, in connection with the fecal pellets, expelled from the mussel’s digestive system (Harris 1993). However, carbon mineralization rates of fresh biodeposits increase considerably after an initial lag phase of 1 day (Carlsson et al. 2010), suggesting bacterial growth and/or colonization by outside bacteria during the lag phase (Canfield et al. 2005). Indeed, Fabiano et al. (1994) showed that bacterial numbers increased during the first 2 days of biodeposit decomposition. However, when biodeposits were added to benthic substrates, maximum levels of carbon mineralization were observed immediately after addition (Giles and Pilditch 2006; Carlsson et al. 2010), presumably as a consequence of high microbial biomass in sediments facilitating rapid colonization of the biodeposits. Bacterial colonization, and thus mineralization dynamics, seems to vary between benthic (with substrate) and pelagic (without substrate) systems. Bacterial growth is also dependent on nutrient composition of the biodeposits (del Giorgio and Cole 1998), which in turn depends on the concentration and type of diet of the mussels (Miller et al. 2002; Giles and Pilditch 2006). Furthermore, bacterial growth rates and organic matter decomposition are positively correlated with temperature (White et al. 1991; Katterer et al. 1998). Due to variations in biodeposit composition and fluctuating water temperatures, mineralization rates of decomposing biodeposits may vary over temporal and spatial scales.

The few studies defining mineralization rates for mussel biodeposits did not include temporal or spatial variability (Giles and Pilditch 2006; Carlsson et al. 2010) nor has any study defined the specific effects of temperature or biodeposit composition on biodeposit mineralization rates. The variability in specific mineralization rates is therefore unknown. The current study investigates the difference in mineralization rates of mussel biodeposits during three seasons (March, August and November). Given the fact that phytoplankton dynamics are influenced both by nutrient availability and stoichiometry (Redfield ratio; Redfield et al. 1963), a multiple-element approach was applied to the present study, and mineralization rates were determined for carbon (C), nitrogen (N), phosphorus (P) and silicate (Si). The study was carried out in a fjord area in South-West Norway, which is characterized by oligotrophic conditions (Jansen et al. 2011) and relatively low water temperatures (between 4 and 18 °C on an annual basis; Sætre 2007). The oligotrophic conditions in our study area were different from the studies by Carlsson et al. (2010) and Giles and Pilditch (2006), who investigated more eutrophic conditions. As the trophic conditions determine the food quality and quantity for the mussels, it might also influence the nutrient composition of the produced biodeposits and thus remineralization rates. The physical conditions of most fjord systems (deep stratified water columns) inhibit the contribution of benthic remineralized nutrients to the nutrient pool in the euphotic zone (Aure et al. 1996; Asplin et al. 1999). Biodeposit mineralization in the pelagic phase is therefore the most important decomposition site in the context of fjord-mussel culture interactions (Jansen et al. 2012). Biodeposits produced by the mussels are partly trapped in between the mussel matrix of suspended ropes (Jansen et al. 2011), and its decomposition contributes to nutrient regeneration in the pelagic zone (Richard et al. 2006). To mimic continuous deposition on suspended ropes, biodeposits were added to incubation chambers on a daily basis during an experimental period of 3 weeks. Since the labile nutrient pool in biodeposits becomes depleted within several days (Giles and Pilditch 2006; Carlsson et al. 2010), we expected to observe stable nutrient release rates toward the end of the experimental period. The relatively long experimental period was chosen, so that a stable microbial community could develop, as is the case for in situ ropes, and to cover variability in stable state estimates reflecting fluctuations in environmental conditions. This experimental approach allowed us to test the following hypothesizes: (1) higher biodeposit nutrient composition will lead to higher mineralization rates, (2) mineralization rates expressed as fraction of the available organic nutrient in the biodeposits (e.g., CO_2_ released/POC in biodeposit) will be similar between temperatures, and (3) respiration and nutrient releases will reach stable state conditions more rapidly at higher temperatures.

## Materials and methods

### Experimental design

The study was carried out at Austevoll Research Station that is situated in a fjord area in South-West Norway (N60°05′, E005°16′). The laboratory facilities received unfiltered seawater (1.5 m depth) that was pumped into two header tanks (400 l). The estimated resident time in the header tanks was less than 0.2 h, and hence, environmental conditions were assumed to be similar to natural conditions.

Respiration and nutrient release rates from decomposing biodeposits were quantified for three seasons with varying food concentrations and water temperatures: spring (March), summer (August) and autumn (November). During each of the seasons, freshly collected mussel biodeposits were added daily to incubation chambers during an experimental period of 3 weeks. Respiration and nutrient release rates from decomposing biodeposits were determined every 2nd day throughout the experimental periods.

### Biodeposit collection

The approximately 750 mussels *Mytilus edulis* (1–2 year old) used for the experiments were kept outdoor in cages (1.5 m depth) in between the experimental periods. Mussels were acclimatized to the laboratory conditions 1 week prior to the first biodeposit collections. The mussels were randomly divided over three biodeposit collection tanks (*V* = 50 l; Ø = 50 cm; *H* = 25 cm), in which they were placed on a tray at mid-water depth. The tanks received seawater through an inflow opening at the bottom of the tank (15 l min^−1^) and a drain along the entire top of the tank served as the outflow. This upwelling design assured a well-mixed water column providing food and oxygen to the mussels, while the biodeposits sank to the bottom of the tank. Water quality parameters describing mussel food quantity and quality are presented in (Jansen et al. 2011, 2012). Biodeposits were collected once a day with syringes, and remaining biodeposits were removed after each collection, assuring that all collected biodeposits were not older than 24 h.

Biodeposits were collected in excess and were used for the following three purposes: (1) for measurements on mineralization rates, (2) to monitor the amount of biodeposits added to each chamber and (3) to determine the quality of the biodeposits. A volumetric approach was used for standardizing the amount of biodeposits added to the incubation chambers. To monitor the amount of biodeposits added to the incubation chambers, triplicate samples, with a similar volume as added to the chambers, were filtered onto pre-weight filters (Whatman GF/A) and salt was expelled by rinsing each filter with deionized water. Filters were dried at 60 °C for at least 12 h to determine dry weight (DW) and combusted at 450 °C for 6 h to determine ash-free dry weights (AFDW). Another biodeposit sample was collected for the determination of biodeposit quality. These samples were pooled into weekly samples, resulting in 3 samples (week I, II and III) per experimental period. Following the experimental period of 3 weeks, the remaining biodeposits were collected from the incubation chambers. Both the fresh biodeposit samples and post-mineralization samples were dried and homogenized, and a subsample was analyzed using a Thermo Finnigan Flash EA 1112 NC Analyzer to determine organic carbon and nitrogen composition. To determine organic phosphorus, a subsample was analyzed using spectrophotometric methods as described by Grasshoff et al. (1999).

### Mineralization measurements

The experimental unit for mineralization measurements consisted of six incubation chambers placed in a water-bath with running seawater ensuring that temperatures were similar to ambient values. Biodeposits were added to 4 of the incubation chambers, and the remaining two chambers were used as a control. The incubation chambers consisted of 1.2-l sealed tanks (Ø = 10 cm; *H* = 13 cm) with the water inflow tap located at mid-water depth and a water outflow tap positioned at the top of the chamber. Water flow through the chambers was set to 180–200 ml min^−1^, and incubations were performed by closing the in- and outflow taps. Magnetically driven stirring bars fitted into the inside of the lids mixed the water in the chambers during the incubations. A dye test confirmed that the water column was well-mixed during both the incubations and flow periods, while biodeposit resuspension was prevented. Incubations were terminated when the oxygen concentration had decreased approximately 10 % compared to initial values, resulting in incubation times ranging from 2 to 12 h. A linear oxygen decline through time was confirmed by a pilot study (data not shown), where an oxygen optode with a measurement interval of 1 s was mounted inside a chamber during the incubation period. All incubations were performed in the dark to limit absorption of nutrients by phytoplankton. Oxygen measurements (optode no. 4835, Aanderaa) and water samples for dissolved inorganic nutrient concentrations were taken in all chambers at the start and end of each incubation. Samples (total 20 ml) for nitrite, nitrate, phosphate and silicate were preserved with chloroform and stored in a cool and dark place until analysis. Those samples were analyzed according to standard methods (Parson et al. 1992) adapted for an auto-analyzer. Total ammonia nitrogen (TAN) samples (20 ml) were directly frozen until analysis. TAN concentrations were analyzed by means of fluorometric analysis (Kerouel and Aminot 1997; Holmes et al. 1999).

### Data standardization and statistical analysis

Prior to statistical analysis, all data were checked for homogeneity and normality of variance assumptions by (1) visually examining standardized residuals versus predicted values plots and Q-Q plots of residuals, (2) Shapiro-Wilk tests and (3) Levene tests (Quinn and Keough 2002). All statistical analyses were performed using SAS 9.2, and data are presented as mean ± standard error (SE), unless stated otherwise.

One-way analysis of variances (ANOVA) tests were used to test variability in biodeposit quality (OM, POC, PON, POP) between the three experimental periods for both freshly collected biodeposits and biodeposit remnants from the incubation chambers at the end of the experimental period. In case of significant results, Tukey’s HSD post hoc multiple comparison tests were used to determine which of the experimental periods were significantly different from each other.

Oxygen concentrations were recalculated into carbon (CO_2_) concentrations assuming a respiratory quotient (RQ) of 1 (Findlay et al. 1986; Hatcher et al. 1994; Tornblom and Bostrom 1995). Nutrient release rates of decomposing biodeposits were calculated according to the following equation:

1 $$ N = \frac{{({\text{NC}}_{\text{end}} - {\text{NC}}_{\text{start}} ) \times V}}{t} $$

where *N* is nutrient release rate (μmol day^−1^ for CO_2_, TAN, PO_4_ and Si(OH)_4_), NC is nutrient concentration at start and end of each incubation (μmol l^−1^), *V* is the total volume of the incubation chamber (1.2 l), and *t* is incubation time (day). Rates were corrected for the control measurements, although fluxes in the control chambers fluctuated around zero for the majority of the measurements. Broken line analysis was used to estimate timing and amplitude of the stable state conditions for respiration and nutrient releases (Koops and Grossman 1993; Robbins et al. 2005). The analysis was performed with the nonlinear regressions (PROC NLIN) using the following model statement:

2 $$ N = A - S \times Z $$

This model fits a diphasic function with a one slope linear function and an upper asymptote, where *N* represents the nutrient flux (in μmol day^−1^ for CO_2_, TAN, PO_4_ or Si), *A* is the asymptote representing the stable state conditions, *S* is the slope of the linear function, and *Z* was defined as ((day < *B*) ∗ (*B* − day)) which indicates that (*B* − day) is defined as zero if (day > *B*). This allows us to estimate the inflection point (*B*) that represents the day when the stable state conditions were reached. Estimates of *B* were restricted by bound statements that were set to the duration of the experimental period (*B* < 20 for March; *B* < 22 for August and November). Subsequently, estimates for *A*, *S* and *B* were obtained by iterative processes. One-way ANOVA was performed to test the difference in *B* values between experimental periods.

Nutrient stoichiometry of C:N, N:P and N:Si for release rates was calculated in atomic equivalents. Two-way multivariate analysis of variance (MANOVA) was used to test the effect of day and experimental period on the complete dataset.

## Results

### Temperature profiles

**Fig. 1:**
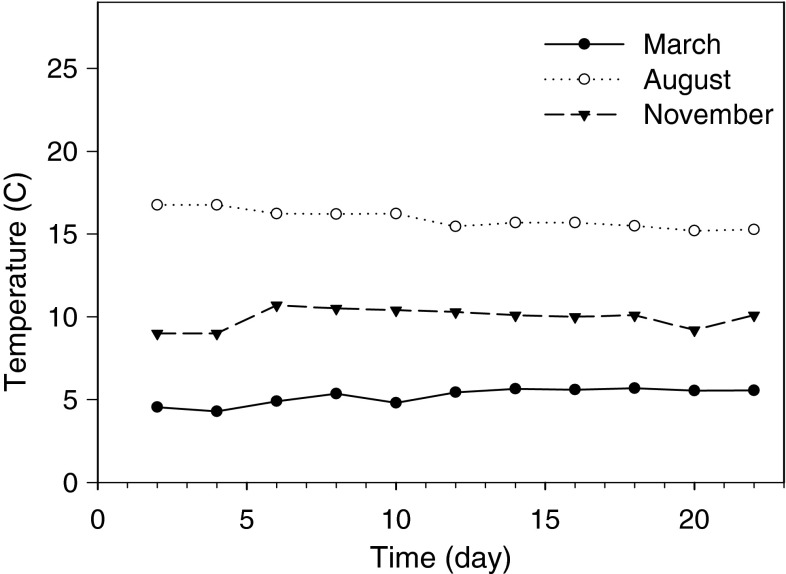
Temperature profiles measured in the incubation chambers during the three experimental periods (March, August and November)

The experimental periods were significantly different from each other (Tukey HSD; *p* < 0.05) with approximately 5 °C differences between them (Fig. 1). Lowest temperatures were observed in March (5.2 ± 0.5 °C), highest temperatures in August (15.9 ± 0.6 °C) and intermediate temperatures in November (9.9 ± 0.6 °C).

### Biodeposit characteristics

**Fig. 2:**
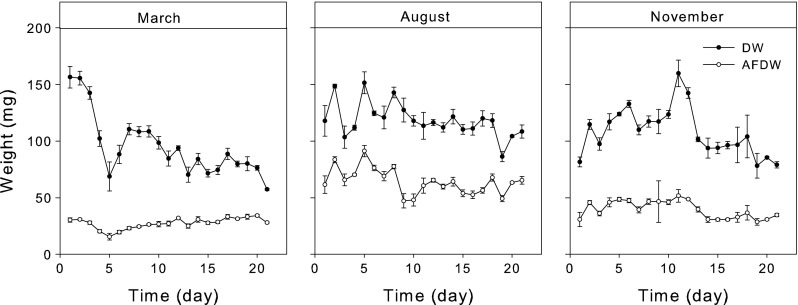
Average daily biodeposit quantity, in dry weight (DW) and ash-free dry weight (AFDW), added to the incubation chambers during the three experimental periods (March, August and November)

Variations in the quantity of biodeposits added to the incubation chambers were observed over both daily and seasonal time scales (Fig. 2). On average, 95.3 ± 6.0 mg, 118.5 ± 3.3 mg and 107.9 ± 4.6 mg biodeposits were daily added to the incubations chambers in March, August and November, respectively. The organic fraction of the biodeposits varied considerable during the March experiment, from 19 % in the first days of the experiment to >45 % during the last days. The overall organic fraction was thereby significantly lower for the March experiment (28.8 ± 2.1 %) compared to August (54.2 ± 1.5 %) and November (36.5 ± 0.7 %; Tukey HSD; *p* < 0.05; Table 1). Pseudofeces was only produced during the first days of the March experiment (visual observations), which coincided with the spring bloom. Carbon and nitrogen concentrations were highest in August and lowest in March, although there were no statistical significant differences between the experimental periods (ANOVA; *F*_2,8_ = 1.83, *p* = 0.240 for carbon and *F*_2,8_ = 1.93, *p* = 0.226 for nitrogen). Phosphorus concentrations were highest in March and lowest in November (ANOVA; *F*_2,8_ = 5.56, *p* = 0.043). CN ratios were stable both on weekly as well as seasonal time scales (CN = 10.6), whereas NP ratios showed more variation (from 11.4 to 41.7) with significantly different values between November and March (Tukey HSD; *p* < 0.05).

**Table 1:** Quality characteristics of freshly deposited biodeposits

	OM	POC	PON	POP	CN	NP
March						
Week I	20	62.0	6.9	1.3	10.4	11.4
Week II	30	104.1	11.3	1.6	10.7	16.0
Week III	41	139.1	15.1	1.6	10.7	20.8
Average	29 ± 2^a^	101.7 ± 22.3^a^	11.1 ± 2.4^a^	1.5 ± 0.1^a^	10.6 ± 0.1^a^	16.1 ± 2.7^a^
August						
Week I	59	101.1	10.8	1.1	10.9	21.3
Week II	50	189.0	20.4	1.3	10.8	33.6
Week III	54	194.0	22.9	1.7	9.9	30.6
Average	54 ± 2^b^	161.4 ± 30.2^a^	18.0 ± 3.7^a^	1.4 ± 0.2^ab^	10.5 ± 0.3^a^	28.5 ± 3.7^ab^
November						
Week I	38	125.6	15.5	1.1	9.4	30.3
Week II	36	140.7	15.5	0.8	10.6	41.7
Week III	36	166.1	16.6	1.0	11.7	36.1
Average	37 ± 1^c^	144.1 ± 11.8^a^	15.9 ± 0.4^a^	1.0 ± 0.1^b^	10.6 ± 0.6^a^	36.0 ± 3.3^b^

Data are expressed as the organic fraction (OM; %), particulate organic carbon (POC), nitrogen (PON) and phosphorus (POP) composition (mg element g^−1^ DW) and molar CN and NP ratios

Different letters indicate significant differences (*p* < 0.05)

Carbon and nitrogen concentrations were 2–4 times lower at the end of the experiments compared to the fresh biodeposits (Table 2). However, phosphorus concentration of biodeposits present in the chambers at the end of the experiment decreased less than for carbon and nitrogen and was only 1.1–1.7 times lower compared to POP concentrations in fresh biodeposits. CN ratios in March and August decreased during decomposition, while elevated values were observed in November. NP ratios in the biodeposit remnants were substantially lower compared to ratios in fresh biodeposits for all three experimental periods.

**Table 2:** Quality characteristics of partly decomposed biodeposits at the end of the experimental period

		POC	PON	POP	CN	NP
March		41.5 ± 3.9	5.4 ± 0.5	0.88 ± 0.06	9.0 ± 0.1	13.7 ± 1.5
August		59.1 ± 6.7	7.2 ± 0.9	1.32 ± 0.13	9.6 ± 0.2	12.6 ± 2.3
November		37.3 ± 1.7	3.6 ± 0.2	0.78 ± 0.02	12.1 ± 0.3	10.3 ± 0.5

Data are expressed as the average (±SE) particulate organic carbon (POC), nitrogen (PON) and phosphorus (POP) composition (mg element g^−1^ DW) and molar CN and NP ratios

### CO_2_ and nutrient release rates

**Fig. 3:**
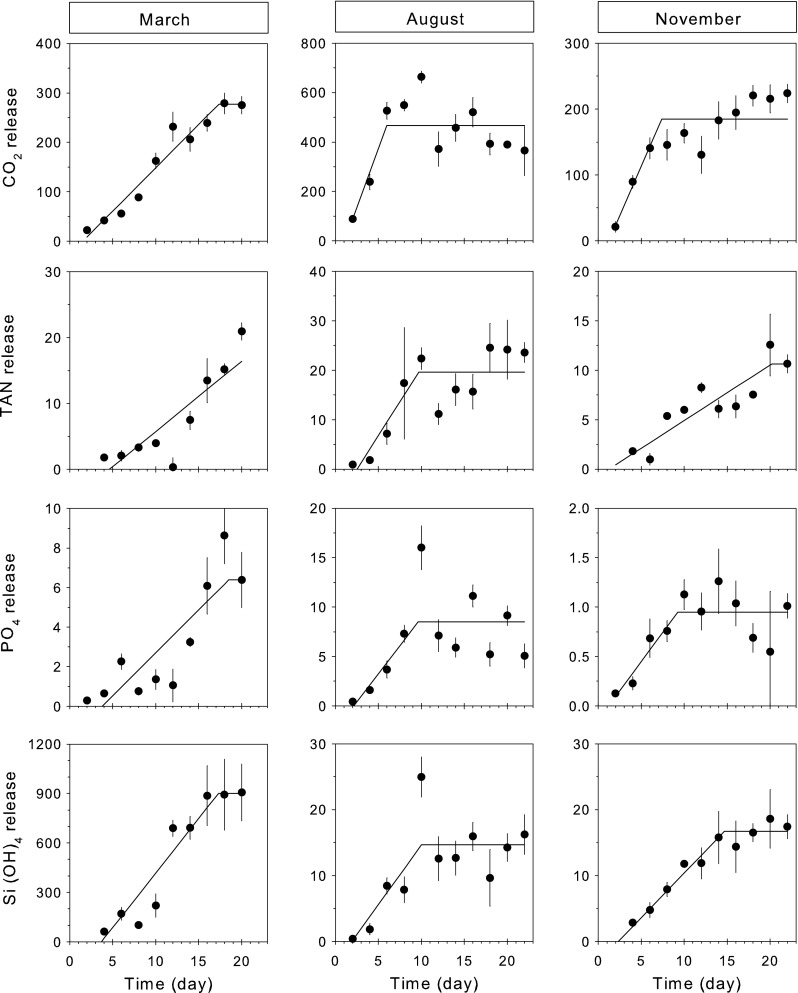
Measured (*dots*; mean ± SE) and modeled (*solid line*) release of carbon dioxide (**a**), total ammonia nitrogen (**b**), phosphate (**c**) and silicate (**d**) from decomposing mussel biodeposits during the three experimental periods (March, August and November). Modeled values are based on the *broken-line* analysis (see also Table 3)

During all three experimental periods, a steep increase in CO_2_ TAN, PO_4_ and Si(OH)_4_ releases was observed from the total amount of biodeposits present in the mineralization chambers (Fig. 3). The steep increases were followed by a stable state condition where releases were relatively constant, indicating that the labile nutrient pool of biodeposits added to the chambers at day 1 becomes depleted. The timing when stable state conditions were reached (inflection point *B*) varied between experimental periods (Table 3; ANOVA; *F*_2,9_ = 6.98; *p* < 0.05). Overall stable state conditions were reached after 18.3 days in March, 8.8 days in August and 12.9 days in November. During all three periods, CO_2_ reached stable state conditions before the other nutrients (TAN, PO_4_ and Si(OH)_4_). Releases during stable state, indicated by the asymptote (*A*), also varied between experimental periods (Table 3). The most striking difference was found for the silicate releases in March, which were 60-fold higher than for August and November measurements. Nitrogen release was dominated by TAN releases, and nitrate and nitrite concentrations or fluxes were below the detection or sensitivity limits of the instrument for many sampling points (data not shown). Copepods settled in the incubation chambers during the last 2 days of the March experiment, which resulted in high oxygen consumption and TAN excretion rates that could not be related to biodeposit composition but rather were an effect of copepod metabolism. Results of the last sampling in March (day = 22) were therefore not included into the analysis.

**Table 3:** Results of the broken line analysis for nutrient release rates (N) of decomposing biodeposits during the three experimental periods

Experimental period	Variable (N)	A	S	B	*F*	*p*
March	CO_2_	276.9 ± 13.0	17.5 ± 1.4	17.3 ± 1.1	125.7	<0.0001
	TAN	16.4 ± 1.2	1.1 ± 0.1	19.3 ± 7.1	90.0	<0.0001
	PO_4_	6.4 ± 1.1	0.4 ± 0.1	18.5 ± 2.9	25.1	<0.0001
	Si(OH)_4_	900.0 ± 80.6	66.1 ± 8.8	17.3 ± 1.8	46.7	<0.0001
August	CO_2_	466.1 ± 21.2	94.7 ± 44.0	6.0 ± 1.5	20.2	<0.0001
	TAN	19.6 ± 1.7	2.7 ± 1.0	9.7 ± 2.1	13.4	<0.0001
	PO_4_	8.5 ± 0.7	1.1 ± 0.4	9.6 ± 2.0	13.9	<0.0001
	Si(OH)_4_	14.7 ± 1.1	1.8 ± 0.7	10.0 ± 2.1	17.5	<0.0001
November	CO_2_	184.5 ± 8.2	29.9 ± 8.2	7.4 ± 1.1	27.4	<0.0001
	TAN	10.6 ± 1.3	0.6 ± 0.1	20.2 ± 2.6	40.3	<0.0001
	PO_4_	1.0 ± 0.1	0.1 ± 0.1	9.2 ± 2.4	7.6	<0.01
	Si(OH)_4_	16.7 ± 1.1	1.4 ± 0.2	14.7 ± 1.5	40.5	<0.0001

Estimates (±SE) of the asymptote (A), slope (S) and inflection point (B), and *F* and *p* statistics for the diphasic function

*N* = *A* − *S * * *Z* (with *Z* = (day < *B*) − (*B* − day))

**Fig. 4:**
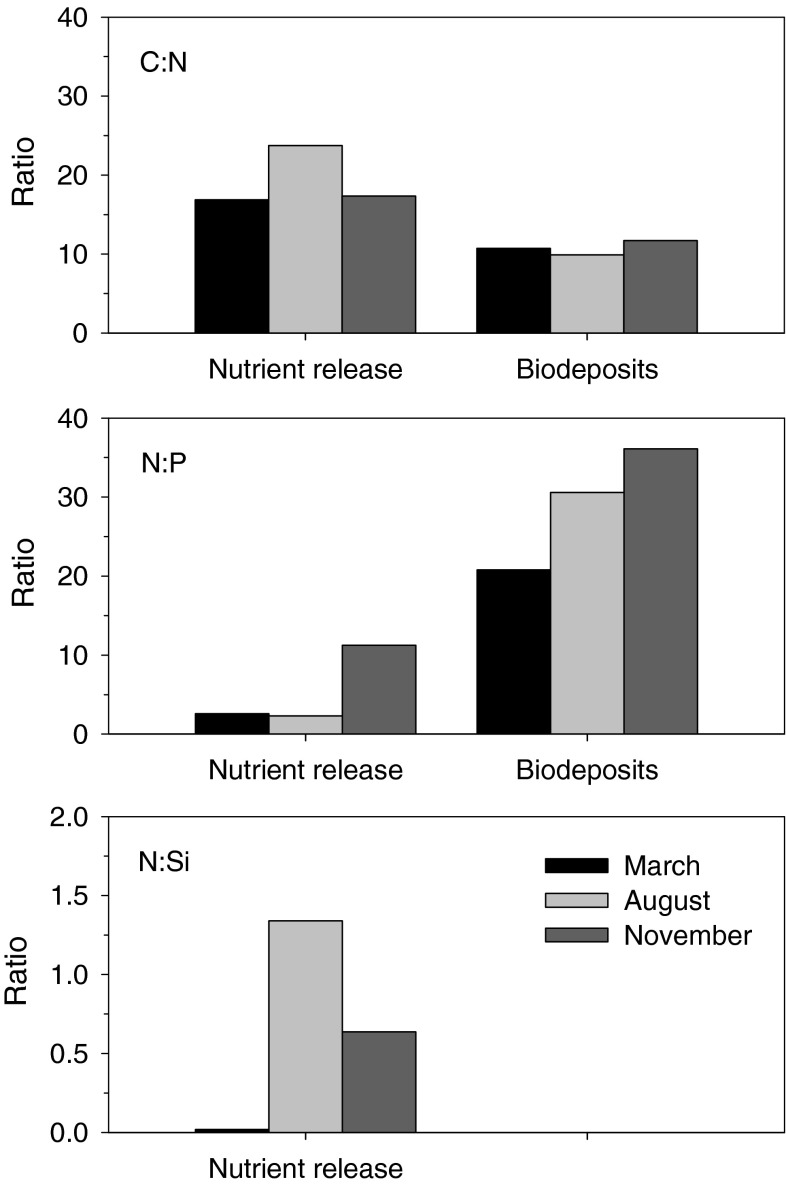
Stoichiometric comparisons (CN, NP and NSi) of nutrient release rates from decomposing mussel biodeposits and for organic nutrient composition in fresh mussel biodeposits for the three experimental periods (with *black squares* for March, *light grey squares* for August, and *dark grey squares* for November) during stable state conditions

Stoichiometric comparisons (CN, NP and NSi release ratios) varied both within and between experimental periods (MANOVA; *p* < 0.05). Likewise, overall the nutrient stoichiometry was different between the three experimental periods during the stable state conditions (Fig. 4). CN ratios in August (~23) were higher compared the CN values in March and November (~17), and NP ratios in November (~11) were higher than March and August (~2.5). NSi ratios were highest in August (1.3), mediocre in November (0.6) and lowest in March (0.02). For each experimental period, we observed CN flux ratios that were higher compared to CN ratios in the biodeposits, and NP flux ratios that were lower compared to NP ratios in the mussel biodeposits.

### Mineralization rates

Mineralization rates were determined by correcting the stable state estimates for CO_2_, TAN, PO_4_ and Si(OH)_4_ (defined by the asymptote *A* in the broken line analysis, Table 3) for average daily biodeposit quantity (DW) to obtain weight standardized mineralization rates (upper section Table 4). Subsequently, mineralization was standardized to organic nutrient concentrations in the biodeposits (POC, PON and POP) to obtain mineralization rates corrected for biodeposit quality (lower section Table 4). However, phosphate mineralization rates in March and August exceeded potentially feasible values based on biodeposit nutrient composition, indicated by fractions >100 % when mineralization rates were standardized to the fraction of initial nutrient composition (lower part Table 4; values within parentheses). High silicate releases in March lead to mineralization rates that were almost 60–90-fold the values in August and November. Due to the absence of estimates for biogenic silicon in mussel biodeposits, it was not possible to derive Si mineralization rates standardized to element quantity in the biodeposits. On average, 24 ± 5 % of the carbon and 17 ± 3 % of the nitrogen initially available in mussel biodeposits are mineralized, with lower values observed in November compared to March and August.

**Table 4:** Mineralization rates derived from the stable state measurements during the three experimental periods (March, August and November)

	CO_2_	TAN	PO_4_	Si(OH)_4_
Mineralization (mmol g^−1^ DW biodeposit day^−1^)				
March	3.53	0.21	0.08	11.47
August	4.30	0.18	0.08	0.14
November	2.04	0.12	0.01	0.18
Annual average	3.29 ± 0.66	0.17 ± 0.03	0.06 ± 0.02	3.93 ± 3.77
Mineralization (% of the organic nutrient composition in biodeposits)				
March	31	20	(157)	
August	27	11	(147)	
November	15	10	32	
Annual average	24 ± 5	17 ± 3		

Total nutrient degradation of the biodeposits added to the incubation chambers was quantified in two ways. In the first approach (I), release rates of the inorganic nutrients were integrated from day 1 to day 22, which provided total release rates for the entire experimental period, and were compared to the total amount of organic nutrients added—with biodeposits—to the incubation chambers. This indicated that on average 22 ± 5 % carbon and 11 ± 3 % nitrogen were mineralized during the entire experimental period (Table 5). Phosphorus mineralization showed large fluctuations between the three experimental periods, with flux estimates outreaching the potential values based on biodeposit phosphorus concentration in August (>100 %). In the second approach (II), decomposition estimates were based on nutrient composition in fresh biodeposits and biodeposit remnants present in chambers at the end of the experimental period. Initial concentrations showed 3–5 times higher values, indicating that on average 66 ± 4 % carbon and 63 ± 8 % nitrogen were removed from the biodeposits during the experimental period. Based on inorganic nutrient fluxes (approach I), lower degradation estimates were observed for carbon and nitrogen compared to degradation based on organic nutrient composition in biodeposit remnants (approach II). Patterns observed for phosphorus were opposite from carbon and nitrogen, with higher degradation estimates based on approach I.

**Table 5:** Nutrient degradation as a fraction (%) of initial nutrient composition based on two complementary approaches: (I) integrated nutrient fluxes related to the total amount of nutrients added to the incubation chambers during the whole experimental period and (II) nutrient composition in the biodeposit remnants in the incubation chambers following the experimental period related to nutrient composition in fresh biodeposits

	Carbon		Nitrogen		Phosphorus	
	Flux	Composition	Flux	Composition	Flux	Composition
March	25	59	15	52	82	41
August	28	63	10	60	133	4
November	13	74	5	77	23	21

## Discussion

This study presents mineralization rates for biodeposits trapped in the pelagic phase, for example in between the mussel matrix of suspended cultures (Richard et al. 2006; Jansen et al. 2011), and we showed that up to one-third of the organic nutrients in biodeposits can be mineralized and thereby become available for primary producers. Although it is well established that degradation of mussel biodeposits leads to enhanced respiration and release of inorganic nutrients, little has been published on biodeposit quality and their specific mineralization rates (reviewed by McKindsey et al. 2011). Hence, our study contributes with new data and insights to this field. Mineralization rates specific for biodeposits are a necessity for optimizing models describing the contribution of bivalves in nutrient cycling of coastal ecosystems. Currently, there is a need for more accurate representation of the effects of biodeposit mineralization in existing carrying capacity models (Henderson et al. 2001).

### Mineralization rates

The present study showed that 24 % of the POC and 17 % of the PON in mussel biodeposits is mineralized. This is in the same range as reported by Carlsson et al. (2010) who showed that, respectively, 25 and 38 % of the POC in biodeposits are mineralized during decomposition with and without benthic substrate. Giles and Pilditch (2004) indicated that 40 and 18 % of, respectively, the POC and PON composition in biodeposits became mineralized into CO_2_ and TAN. These authors also suggested that additionally 34 % of the PON might have been removed by bacterial assimilation and/or nitrification-denitrification processes and N_2_ production. As our systems did not include sediment and anoxic conditions were prevented, we assume that in this study N_2_ production was insignificant. However, anoxic microsites within biodeposits, as suggested by Gowing and Silver (1983), could allow denitrification to occur. The abovementioned studies (Giles and Pilditch 2006; Carlsson et al. 2010) evaluated respiration and nutrient release rates through time (4–10 days) after a single addition of mussel biodeposits. This is different from our approach with multiple additions of biodeposits, and we were therefore also able to quantify mineralization rates under steady-state conditions (see Table 4), which represent CO_2_ and nutrient releases under natural conditions (e.g., within a farm) where continuous deposition of biodeposits occurs. An approach with continuous biodeposition was also chosen by Callier et al. (2009) who performed an in situ benthocosm experiment where mussels were placed on top of sediment cores, and sediment respiration and nutrient release rates were measured after a 50-day enrichment period (summer). Recalculating nutrient releases from Callier et al. (2009) based on estimated biodeposition rates, results in maximum mineralization rates of 4.5 mmol CO_2_, 0.3 mmol TAN, 0.02 mmol PO_4_ and 1.0 mmol Si(OH)_4_ per gram biodeposit (DW) per day. CO_2_ mineralization rates were thereby in the same scale as reported for the August results in our study, while TAN and phosphate releases were lower compared to our results. Phosphate releases from sediments underneath suspended bivalve cultures show incoherent results, with positive fluxes measured in some cases (Baudinet et al. 1990; Souchu et al. 2001; Richard et al. 2007b) and neutral or negative fluxes in others (Hatcher et al. 1994; Mazouni et al. 1996; Giles and Pilditch 2006). Low or negative phosphate fluxes are often explained by the absorption of phosphate by iron hydroxides or calcite present in oxidized surface layer of marine sediments (Sundby et al. 1992). Silicate mineralization rates in our study varied between 0.1 and 11.4 mmol g^−1^ day^−1^, which includes the rates reported by Callier et al. (2009). Silicate releases from mussel biodeposit enriched sediments have reported to be in the same order of magnitude or higher than ammonia releases (Baudinet et al. 1990; Richard et al. 2007a, b). Smaal and Prins (1993) also reported relatively high silicate releases from decomposing biodeposits, while no silicate releases from decomposing mussel biodeposits were observed by Fabiano et al. (1994).

Total nutrient mineralization throughout the experimental periods was 3–5 times lower for carbon and nitrogen based on flux measurements compared to biodeposits nutrient composition measurements, indicating that more nutrients were released from the biodeposits than was estimated based on the measured inorganic nutrient fluxes. This may partly be due to the fact that biodeposits were added to the chambers after the incubations, and hence, mineralization during the first day was not included in the measurements. Potentially, this has led to an underestimation of the nutrient fluxes and mineralization rates. However, similar mismatches between inorganic nutrient releases and biodeposit nutrient composition were observed by Carlsson et al. (2010) who related the relatively low nutrient composition in decomposed feces to the release of dissolved organic material. They demonstrated that only 53 % of the organic carbon (POC) degradation in biodeposits could be attributed to mineralization-related processes (CO_2_ release), whereas 47 % was related to leaking of labile dissolved organic carbon (DOC) from the fecal pellets. Dissolved organic nutrients might have been removed from our experimental chambers during the periods in between the incubations when the chambers functioned as a flow-through system; hence, carbon and nitrogen mineralization rates reported for our study may represent an underestimation. Phosphorus results in our study showed an opposite pattern to carbon and nitrogen, as higher degradation values were observed based on the flux measurements compared to biodeposits nutrient composition measurements, indicating that more phosphate was released from decomposing biodeposits than predicted from nutrient composition in the biodeposit remnants at the end of the experimental period. This was particularly clear in August when flux estimates exceeded the theoretical value of 100 % and POP composition in the biodeposit remnants was comparable to the composition in fresh biodeposits. This suggests that potentially there has been an external source of phosphorus accumulating onto, or binding with, the biodeposits throughout the experimental period, which partially reversed during the incubations. Further research is required to determine the potential mechanisms that underlie to these processes.

### Mineralization kinetics and seasonal variability

Mineralization kinetics vary between the different elements and are influenced by seasonal changes in temperature and biodeposit nutrient composition. Carbon, nitrogen and phosphorus are mineralized by microbial activity, whereas silicate mineralization is essentially different as it relies on dissolution processes rather than biological activity (Canfield et al. 2005). Carbon mineralization was faster compared to the other elements, indicated by lowest inflection points (B) during all experimental periods. This is in agreement with Giles and Pilditch (2006) who showed that maximum respiration (CO_2_ release) occurred before maximum TAN releases. It is generally assumed that silicon in biodeposits is mineralized on long time scales (Baudinet et al. 1990; Nelson et al. 1995). However, during all three experimental periods, we observed an immediate response in silicate releases. Although underlying kinetics may vary between the different elements, mineralization processes of all four elements (C-N-P-Si) should be positively correlated with temperature as both bacterial growth and dissolution processes are enhanced by increasing water temperatures (Lerat et al. 1990; White et al. 1991; Katterer et al. 1998). Indeed we observed faster mineralization at increasing temperatures, with a 2–3 times higher inflection point (*B*) in spring compared to summer. This fits with *Q*_*10*_ values that are typically assumed to vary between 2 and 3 (Katterer et al. 1998; Thamdrup and Fleischer 1998), indicating that organic matter decomposition increases by a factor 2–3 with a 10 °C increase in temperature.

Biodeposits are rich in nutrients, and organic nutrient concentrations and stoichiometry are essential rate-controlling parameters for mineralization (del Giorgio and Cole 1998; Canfield et al. 2005). Nutrient composition varies on both temporal and spatial scales (Kautsky and Evans 1987; Jaramillo et al. 1992; Giles and Pilditch 2004) and is influenced by the quantity and quality of food the mussels feed on (Miller et al. 2002; Giles and Pilditch 2006). Our study area is characterized by low-food conditions throughout most of the year, with exception of phytoplankton blooms in spring and occasionally in autumn (Strohmeier et al. 2009; Jansen et al. 2012). Pseudofeces production is rare in areas with low seston conditions (Strohmeier 2009) and was only observed in the first week of the March experiment during the spring bloom. The biodeposit nutrient composition was relatively low during the first week of March, which is in agreement with Smaal and Prins (1993) who showed that nutrient composition of feces is higher than for pseudofeces. However, this does not seem to be a consisted pattern as other studies reported the opposite with higher nutrient composition for pseudofeces compared to feces (Navarro and Thompson 1997; Giles et al. 2006). In accordance with the lower nutrient composition of pseudofeces, Smaal and Prins (1993) also demonstrated that mineralization rates of pseudofeces were lower compared to feces. Biodeposit mineralization rates measured in our study were an integrated result of feces and pseudofeces mineralization. Indeed, we observed relatively slow increase in TAN and PO_4_ releases during the first week of the March experiment, which might be the result of the presence of pseudofeces. Although the average carbon and nitrogen composition in biodeposits did not vary between seasons, mineralization rates in spring were almost double the rates measured in autumn, suggesting that biodeposits consisted of more labile material during spring season. Additionally, low availability of micronutrients might be limiting mineralization processes in autumn (Dixon 2008), while the abundance of micronutrients is generally high following the winter season. Nutrient concentrations measured in our study were in the higher range of values reported for other areas which ranged between 25–130 and 3–11 mg g^−1^ for POC and PON, respectively (Kautsky and Evans 1987; Jaramillo et al. 1992; Hatcher et al. 1994; Navarro and Thompson 1997; Giles and Pilditch 2004, 2006), indicating that weight (DW) standardized mineralization rates measured in our study will likely be higher from other areas. In order to determine mineralization rates standardized to biodeposit quality, it is necessary to know the biodeposit nutrient composition. However, in general, there is little information available about phosphorus and silicon composition in bivalve biodeposits. Phosphorus composition as measured in our study varied between seasons, and the overall values were lower compared to values reported by Kautsky and Evans (1987). The relatively high phosphate mineralization rates measured in spring and summer compared to autumn are likely the cause of a combination of biodeposit composition and release of phosphate by and external source (see previous section), as standardization to biodeposit quality showed that mineralization rates exceeded potential rates based on phosphorus composition in the biodeposits during spring and summer. Silicate mineralization rates were 60–80 times higher in spring compared to summer and autumn measurements, which might be induced by higher concentrations of biogenic silicon in the biodeposits during spring. Although direct estimates were not available, it is likely that silicon composition of the biodeposits was higher in spring as the phytoplankton population was characterized by high numbers of the diatom species *Skeletomena* during the spring bloom. During summer and autumn, diatoms were less abundant and the phytoplankton population consisted predominantly of small flagellates (Jansen et al. 2012). As diatoms contain high concentrations of silicon while mussels have minimal requirements for this element, nearly all ingested silicon will be expelled with the biodeposits (Wikfors 2011). There is one study providing estimates of biogenic silicon concentrations in mussel (*Modiolus modiolus*) biodeposits (Navarro and Thompson 1997). For this study, conditions were similar to spring bloom conditions at our study site in terms of chlorophyll *a* concentrations, phytoplankton composition (high number of diatoms), and biodeposit carbon and nitrogen composition (Navarro and Thompson 1997; Jansen et al. 2012). They reported a biodeposit silicon concentration that was approximately three times higher than the carbon concentration. Although it is unknown whether biodeposit composition and mineralization rates can be directly linked, it is noteworthy that the C:Si ratio in biodeposits reported by Navarro and Thompson (1997) was similar to the ratio of CO_2_:Si(OH)_4_ in mineralization rates observed in our spring experiment.

### Implications

Through production and mineralization of biodeposits, mussel populations are efficient mediators in nutrient recycling. Biodeposits produced by suspended mussel cultures may decompose at three sites: (1) biodeposits trapped in between the mussel matrix on the suspended mussel ropes decompose in the euphotic zone of the water column where the culture structure is located (Richard et al. 2006), (2) while descending to the seafloor, biodeposits may decompose in the pelagic phase of the water column (Carlsson et al. 2010), and (3) biodeposits reaching the seafloor will either be buried in the sediment or be decomposed by benthic processes (Baudinet et al. 1990; Hatcher et al. 1994). The relative contribution of each of the decomposition sites is situation specific and determined by physical and environmental conditions of the bivalve cultivation area (Newell et al. 2005). In bottom cultures, pelagic decomposition is limited, and it is suggested that benthic biodeposit decomposition contributes significantly to total nutrient regeneration from mussel beds (Asmus et al. 1990; Prins and Smaal 1994). In suspended bivalve cultures, benthic decomposition sites are spatially decoupled from the pelagic culture units (Newell 2004), yet both benthic and pelagic decomposition sites contribute to nutrient regeneration in shallow culture areas due to a strong benthic-pelagic coupling (Prins et al. 1998). However, when benthic-pelagic coupling is limited, as in deep fjord systems, only the decomposition that takes place in the pelagic/euphotic zone is relevant for primary producers.

It is unknown which fraction of the total produced biodeposits is trapped within the mussel matrix and which fraction is transported to the benthic system. Jansen et al. (2011) determined the total amount of organic material associated with mussels rope sections of 1 m length and showed that this was approximately 3 times the total daily biodeposit production per meter rope (recalculated from Jansen et al. 2012). The amount of organic material associated with the mussel ropes was equal between culture sites that were comparable in terms of mussel size and density (Jansen unpublished data), indicating that settling of organic material (biodeposits) on the ropes is space limited. Integrating total organic material on ropes (Jansen et al. 2011) with mineralization rates determined in the current study showed that maximum release rates per meter suspended rope (m^−1^) were in the same order of magnitude with maximum release rates from sediment (m^−2^) underneath suspended mussel farms (Baudinet et al. 1990; Hatcher et al. 1994; Giles et al. 2006; Richard et al. 2007b). However, the pelagic regeneration for suspended ropes is slightly overestimated by this approach as the fraction of inorganic material was lower for mussel biodeposits (Table 1) compared to the material associated with the ropes (Jansen et al. 2011). This suggests that the material on the ropes consists of more refractory material, subsequently leading to lower potential mineralization rates. Lower minimum TAN and phosphate release rates for sediments underneath farms (m^−2^; Baudinet et al. 1990; Hatcher et al. 1994; Giles et al. 2006; Richard et al. 2007b) compared to estimates from the present study for suspended ropes (m^−1^) can partly be related to differences in nutrient release kinetics between benthic and pelagic decomposition sites. Marine sediments may bind phosphate (Sundby et al. 1992) and TAN nitrogen may be transformed into other nitrogenous forms (nitrate, nitrite or nitrogen gas) by nitrification-denitrification processes that occur in oxic/anoxic sediment layer (Canfield et al. 2005; Torres-Beristain et al. 2006), resulting in lower benthic fluxes.

Regenerated nutrients originating from decomposition of biodeposits may enhance the availability of nutrients for primary producers (Prins et al. 1998) but may also affect stoichiometric relations between the elements that potentially can lead to a shift in phytoplankton population composition (Prins et al. 1995). This study showed that mineralization of carbon and phosphorus was preferred over nitrogen, as indicated by higher CN ratios and lower NP ratios for the inorganic nutrient releases compared to the organic nutrient composition of the biodeposits. The relative nitrogen releases (NP and NSi) were below Redfield’s ratios (Redfield et al. 1963) and below ratios measured in the ambient water (Jansen et al. 2011), indicating that regenerated nutrients indeed have different stoichiometric characteristics compared to inorganic nutrient pools available in the natural environment.

## Conclusions

This study has shown that mineralization rates of mussel biodeposits vary seasonally. Variations were induced by the concentrations of (macro-)nutrients (C-N-P-Si) in biodeposits, whereas mineralization rates standardized to biodeposit nutrient composition suggested that the proportion of labile material in the biodeposits or availability of micronutrients also regulates mineralization processes. Increased temperatures enhance biodeposit mineralization with approximately 2–3 times faster turnover at a 10 °C temperature interval (*Q*_*10*_). Furthermore, mineralization dynamics varied between nutrients, with mineralization of carbon and phosphorus being preferred over nitrogen. This resulted in different stoichiometric characteristics of the regenerated nutrients compared to inorganic nutrient pools in the natural environment. Data presented by the current study can be used to optimize the biodeposition compartment of models describing the contribution of bivalves in nutrient cycling of coastal ecosystems.
